# Small- and medium-sized rice fields identification in hilly areas using all available sentinel-1/2 images

**DOI:** 10.1186/s13007-024-01142-1

**Published:** 2024-02-04

**Authors:** Lihua Wang, Hao Ma, Yanghua Gao, Shengbo Chen, Songling Yang, Peng Lu, Li Fan, Yumiao Wang

**Affiliations:** 1 Chongqing Institute of Meteorological Sciences, Chongqing, 401147 China; 2 Department of Geography and Spatial Information Techniques, Center for Land and Marine Spatial Utilization and Governance Research, Ningbo University, Ningbo, 315211 China; 3 College of geoexploration science and technology, Jilin university, Changchun, 130026 China

**Keywords:** SAR, Multi spectral instrument, Rice phenological period, Rice standard spectral curve, Spectral similarity vector algorithm

## Abstract

**Background:**

Mastering the spatial distribution and planting area of paddy can provide a scientific basis for monitoring rice production, and planning grain production layout. Previous remote sensing studies on paddy concentrated in the plain areas with large-sized fields, ignored the fact that rice is also widely planted in vast hilly regions. In addition, the land cover types here are diverse, rice fields are characterized by a scattered and fragmented distribution with small- or medium-sized, which pose difficulties for high-precision rice recognition.

**Methods:**

In the paper, we proposed a solution based on Sentinel-1 SAR, Sentinel-2 MSI, DEM, and rice calendar data to focus on the rice fields identification in hilly areas. This solution mainly included the construction of rice feature dataset at four crucial phenological periods, the generation of rice standard spectral curve, and the proposal of spectral similarity algorithm for rice identification.

**Results:**

The solution, integrating topographical and rice phenological characteristics, manifested its effectiveness with overall accuracy exceeding 0.85. Comparing the results with UAV, it presented that rice fields with an area exceeding 400 m^2^ (equivalent to 4 pixels) exhibited a recognition success rate of over 79%, which reached to 89% for fields exceeding 800 m^2^.

**Conclusions:**

The study illustrated that the proposed solution, integrating topographical and rice phenological characteristics, has the capability for charting various rice field sizes with fragmented and dispersed distribution. It also revealed that the synergy of Sentinel-1 SAR and Sentinel-2 MSI data significantly enhanced the recognition ability of rice paddy fields ranging from 400 m^2^ to 2000 m^2^.

## Introduction

In the context of global digitization and informatization, agriculture in developed countries has entered the era of “precision agriculture” [1–3]. The acquisition of crop information is the premise and foundation for the implementation of precision agriculture [4–7]. Rice is one of the important food crops globally, with more than 2 billion people worldwide consuming it as a staple food [8, 9]. Mastering the spatial distribution and planting area of paddy could offer a scientific foundation for rice production status observation, rice yield forecast and evaluation, precision agriculture implementation, and the national grain production layout plan [10–12]. Meanwhile, rice planting information is significant for the water resource monitoring and rational utilization, as well as the impact assessment of human interventions on the atmospheric environment [13, 14].

Traditional rice cropping area surveys are usually accomplished through field mapping and statistical methods [15, 16], which requires a lot of manpower, material resources and time. Also, human interference may lead to inaccurate rice information [17]. Remote sensing has the characteristics of macroscopic and near real-time, which can quickly and accurately obtain the spatial distribution information of rice in a large area [18, 19]. Especially in recent years, with the continuous emergence of new high-spatial, hyperspectral and microwave sensors and the improvement of classification methods, the accuracy and efficiency of remote sensing observation for rice spatial distribution have been greatly improved, making remote sensing an important technology for rice observation, extraction, and mapping [15, 19, 20].

Optical remote sensing has rich spectral information. Various remote sensing indices (Normalized Difference Vegetation Index, NDVI; Enhanced Vegetation Index, EVI; etc.) can be obtained by band operation for rice identification [21, 22]. Existing studies have indicated that during the rice growth cycle, the variation of the rice vegetation index is greater than that of other land types [23–26]. The overall change trend is as follows: (1) In the transplanting stage, the seedlings are short and the vegetation index is low. (2) As the rice enters the greening, tillering, jointing and booting stages, the rice grows rapidly, so the vegetation index presents a rapid upward trend and reaches a peak at the heading stage. (3) In the mature stage, the rice panicle droops, leaves become yellow, and its vegetation index declines. In addition, due to the moisture change particularity of the rice underlying surface, moisture indices (Land Surface Water Index, LSWI; Normalized Difference Water Index, NDWI; etc.) are also often applied for the extraction of rice planting areas [27–29]. However, in optical remote sensing, the spectra mostly interact with rice leaf crowns. The spectral information of rice structure and features under the canopy is easily obstructed by the canopy, resulting in optical images being unable to reflect the spectral features below the leaf canopy [30, 31]. Moreover, the spectral characteristics of rice also have spectral information similarity or consistency with other crops and vegetation, resulting in the misclassification of other land types as rice [17]. Meantime, the mixed pixel phenomenon always appears on medium-low resolution remote sensing images, which makes rice easily confused with the surrounding land types, such as ponds, wetlands, and dry land in the irrigation or rainwater period. After all, optical remote sensing data is subject to the influence of dense cloud layers, rainy and foggy weather, etc., it is difficult to ensure the imaging in the advantageous time of rice remote sensing monitoring, which in turn affects the rice mapping accuracy.

Different from optical remote sensing, SAR imaging is not affected by solar radiation and has the benefits of all-day and all-weather, making SAR an important technology for rice identification, especially in cloudy/rainy rice planting areas such as tropics and subtropics [32–37]. The SAR backscatter characteristics of rice throughout the phenological period are obvious, roughly as follows: (1) During the rice transplanting, the rice paddy is dominated by water, thus specular reflection is the mainly backscatter mechanism, the backscattering intensity is correspondingly low. (2) From the rice turning green to the heading stage, the rice height increases continuously, and the cylindrical structure is obvious. The backscatter mechanism of the rice field transforms to the volume scattering of the plant and the dihedral angle scattering between the water layer and the plant. The backscatter intensity accordingly increases. (3) During the rice mature stage, the ears droop, the leaves tend to be horizontal and orient randomly, thus the backscatter coefficient decrease. The unique SAR backscatter features of the rice phenological stage play a great role in rice identification [14, 34, 38, 39]. However, using SAR data for rice identification and extraction also reveal some flaws, one is the inherent pepper-and-salt noise. Although noise filtering (wavelet transform, Savitzky-Golay time series filtering) [40], super pixel segmentation [41] can be used to reduce the influence of SAR noise, but it still influences the accurate rice identification to some extent [10, 12, 42]. The limitations also include the obvious geometric distortions on SAR images, especially in areas with large terrain fluctuations, presenting as foreshortening, layover, and shadow, which affect the extraction accuracy of rice [23, 43, 44].

In summary, it remains challenging to accurately monitor the rice planting area using single optical or SAR remote sensing technology. Considering the different imaging mechanisms and wavelength ranges of optical and SAR images, different sensors can reflect the physical information of the rice growth process from different angles. Therefore, combining the respective advantages of optics and SAR, deeply mining the characteristics of rice in optical and SAR images, and then constructing a rice identification algorithm for rice extraction has gradually attracted attention [16, 43, 45–47]. Previous studies have shown that the comprehensive utilization of multi-source remote sensing data can fully leverage their complementary advantages, thereby providing more diverse basic data for rice remote sensing monitoring [38, 48, 49]. These data can be used to create the optimal remote sensing feature dataset for rice identification, then to develop suitable classification algorithms for achieving high-precision rice monitoring. However, the above rice identification studies mainly focus on areas with flat terrain, where rice fields are intensive with medium- or large-sized, such as the lower and middle regions of the Mississippi River in the United States [50, 51], the three northeastern provinces of China [52–54], India [55], and the Red River Delta in Vietnam [56]. In China, rice is predominantly cultivated in the Yangtze River basin and its southern regions, which are characterized by numerous hills and mountains [57–61]. Here the land cover types are diverse, the paddy fields are scattered and broken-up with small- or medium-sized, and the paddy shapes are various. Also, in hilly region, the geographical span of rice cultivation is large, with extremely complex natural conditions such as climate, precipitation and topography varying greatly across different regions. The rice growing environment, growing seasons, and farming systems differ significantly from region to region, including the complexity of rice planting systems (single-season rice areas, single-double mixed areas, and double-season rice areas) and rice planting regions (such as the dispersed rice planting, small- or middle-sized rice fields, and fragmented rice paddies), making it difficult to identify and extract rice with timely and high precision in hilly areas.

Therefore, the paper focuses on rice planting in hilly areas, aiming to realize high-precision recognition and mapping of rice fields with fragmented, scattered distribution. The structure of the manuscript is as follows. In Sect. 2, the case study areas and data sources were introduced. In Sect. 3, for paddy fields high-precision extraction in hilly areas, we proposed a solution based on the Sentinel-2 MSI, Sentinel-1 SAR, DEM, and rice calendar data. The core content was the construction of the standard feature curves of rice and the proposal of the Spectral Similarity Vector (SSV) algorithm for rice identification. In Sect. 4, we showed the rice extraction result from our proposed solution. Detailed discussions were presented in Sect. 5, including the solution’s ability to identify rice in patches of different sizes, under different terrain conditions, and from different data sources. The conclusions were in Sect. 6.

## Case study areas and data sources

### Case study areas

**Fig. 1:**
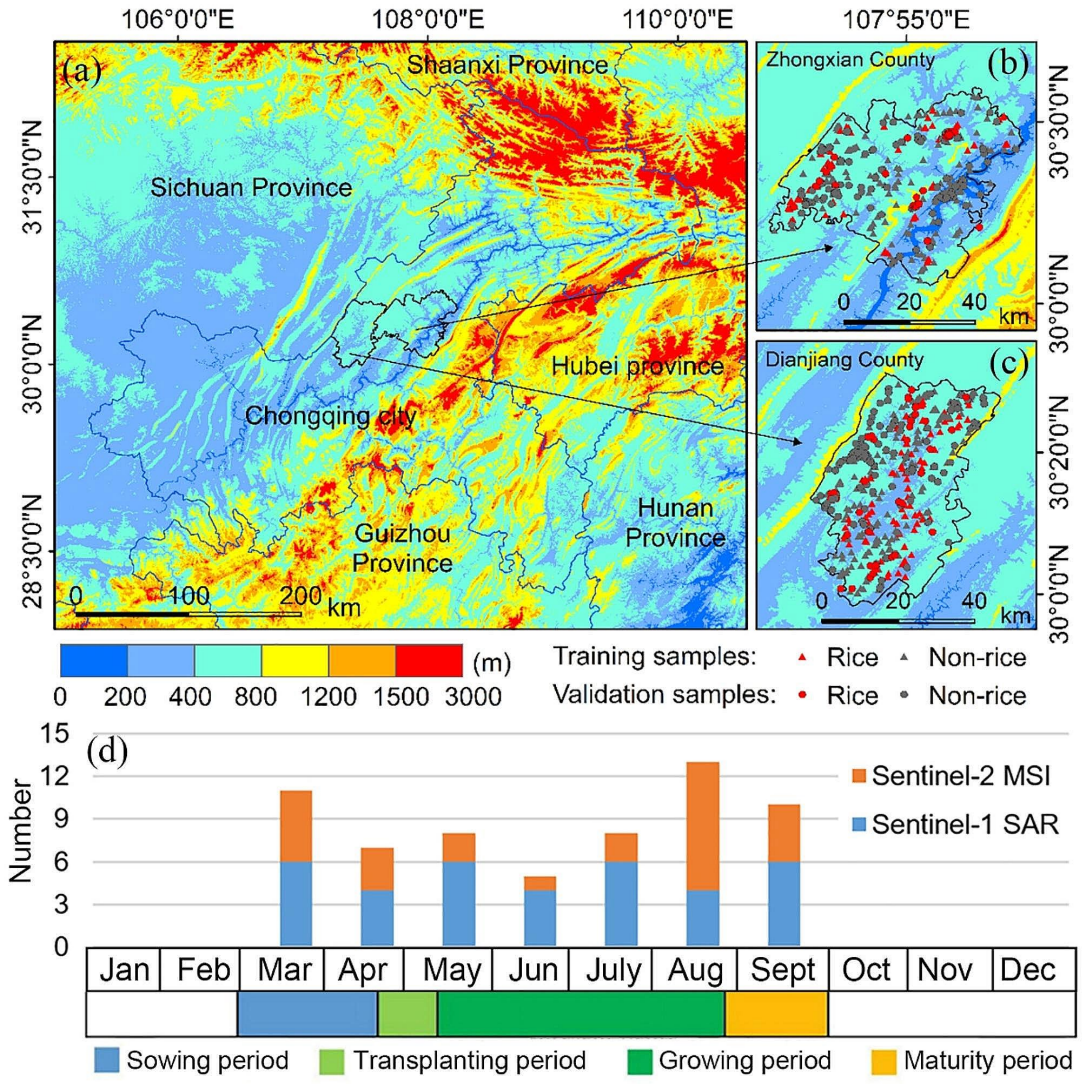
Location and topography of Chongqing, China **(a)**. Topography of Zhongxian County **(b)** and Dianjiang County **(c)** with samples overlaid. Rice phenology in the study area and the number of Sentinel-1 and Sentinel-2 images for each month during the rice phenology period **(d)**

There are six rice-growing regions in China, and three of them are located in the south of China, which account for more than 90% of the country’s rice cultivation area and production (Zhou 1993). Chongqing (Fig. 1a) is a typical single-season rice growing area in the southwest of China, with complex topography (hilly, middle- and low-mountain, and high-mountain areas), complex crop cultivation structure (Rice, vegetables, corn, potatoes, rape, soybeans, peanuts, wheat, sorghum, etc.), large differences in rice patch scales, and fragmented rice patches (36.3% of the rice patch area was below 2000 m^2^) [17].

Zhongxian County and Dianjiang County are located in the central part of Chongqing, with undulating low mountains and intricate streams and rivers. The elevations of Zhongxian and Dianjiang are 117–1680 m and 320–1183 m, respectively, which are typical hilly landforms (Fig. 1b and c). The climate belongs to the subtropical southeast monsoon region mountain climate, with humid air and abundant precipitation (the average annual precipitation of 1175.7 mm). The stereo climate is remarkable with weak solar radiation. The weather is frequent cloudy and foggy.

### Sentinel-2 MSI image and pre-processing

The Sentinel-2 is a wide-swath imaging mission. Its Multispectral Instrument (MSI) sensor has 10 spectral bands, including 4 bands (Blue, Green, Red and NIR) with a high spatial resolution of 10 m and 6 bands (three Vegetation red edge, one Narrow NIR and two SWIR bands) with a spatial resolution of 20 m. We selected all Sentinel-2 MSI images (Level-2 A products) with less than 30% cloudiness covering the case study area (Table 1).

**Table 1:** Detail information of Sentinel-2 MSI data in the paper

Rice phenological stages	Imaging Date
Sowing period	2019/3/10; 2019/3/20; 2019/4/19; 2020/3/19; 2021/2/22; 2021/3/4; 2021/3/24
Transplanting period	2019/4/24; 2019/6/3; 2020/4/28; 2020/5/3; 2020/5/18
Growing period	2019/7/28; 2019/8/2; 2019/8/12; 2019/8/17; 2021/7/22; 2021/8/1
Maturity period	2019/8/22; 2019/8/27; 2020/8/16; 2020/8/26; 2021/8/21; 2021/9/10; 2021/9/20; 2021/9/25; 2021/9/30
Fallow period	2019/12/10; 2020/11/14; 2021/1/13; 2021/2/12; 2021/10/05; 2021/11/24

The pre-processing of Sentinel-2 MSI images includes radiometric calibration, atmospheric correction and cloud mask. The radiometric calibration and atmospheric correction were conducted using Fast Line-of-sight Atmospheric Analysis of Spectral Hypercubes model. The simple cloud score algorithm was used for the cloud mask [62]. The algorithm applies blue, green, red, near-infrared, SWIR1, SWIR2, and the normalized snow index to comprehensively calculate the probability of cloud pollution pixels. Here we chose a threshold > 0.3 for cloud removal.

Due to the influence of topography and climate, Zhongxian and Dianjiang counties have a lot of cloudy weather, resulting in few high-quality Sentinel-2 MSI data to accurately characterize the rice growth cycle. Considering that rice phenological periods in the study area are consistent in adjacent years, in this study, we used Sentinel-2 MSI images from 2019 to 2021 to generate a time series dataset to chart the rice distribution in 2020.

### Sentinel-1 SAR image and pre-processing

The Sentinel-1 A ground-range detected SAR product has a repeat visit rate of 12 days, a high spatial resolution of 5 m in the range direction and 20 m in the azimuth direction. 32 SAR acquisitions with VH and VV polarization in 2020 covering the entire rice planting season were employed (Table 2). These data have been radiometrically calibrated, noise-filtered, terrain-corrected and transformed to decibels via log scaling [17, 63].

**Table 2:** Detail information of Sentinel-1 SAR data in the paper

Rice phenological stages	Imaging Date
Sowing period	2020/2/23; 2020/3/6; 2020/3/18; 2020/3/30; 2020/4/11;
Transplanting period	2020/4/23; 2020/5/5; 2020/5/17; 2020/5/29
Growing period	2020/6/10; 2020/6/22; 2020/7/4; 2020/7/16; 2020/7/28; 2020/8/9;
Maturity period	2020/8/21; 2020/9/2; 2020/9/14; 2020/9/26
Fallow period	2020/1/6; 2020/1/18; 2020/1/30; 2020/2/11; 2020/10/8; 2020/10/20; 2020/11/1; 2020/11/13; 2020/11/25; 2020/12/7; 2020/12/19; 2020/12/31

### Other auxiliary data

Other auxiliary data contain DEM, rice calendar data, Google Earth images and in situ data.

DEM products were generated using Advanced Land Observing Satellite phased-array type L-band SAR Elevation data, which we obtained from NASA Earth Science Data.

Rice calendar data (Fig. 1d) were provided by Chongqing Meteorological Bureau. Its phenological period can be divided into four stages, namely the sowing period (March to mid-April), the transplanting period (mid-late April to early-mid May), the growing period (mid-May to mid-August) and the maturing period (late August to September).

We also conducted field surveys of rice-growing areas in Zhongxian and Dianjiang counties from July 28 to August 4, 2020 and acquired red, green, blue, red-edge, near-infrared band and RGB images at 0.25-m spatial resolution using a DJI Phantom 4 Pro. Finally, we collected 829 samples. Specifically, 363 training samples, including 75 rice samples and 288 non-rice samples, and 186 validation samples, including 65 rice samples and 121 non-rice samples, were collected in Dianjiang County. Zhongxian County had 200 training samples, including 50 rice samples and 150 non-rice samples; and 80 validation samples, including 40 rice samples and 40 non-rice samples.

## Methods

**Fig. 2:**
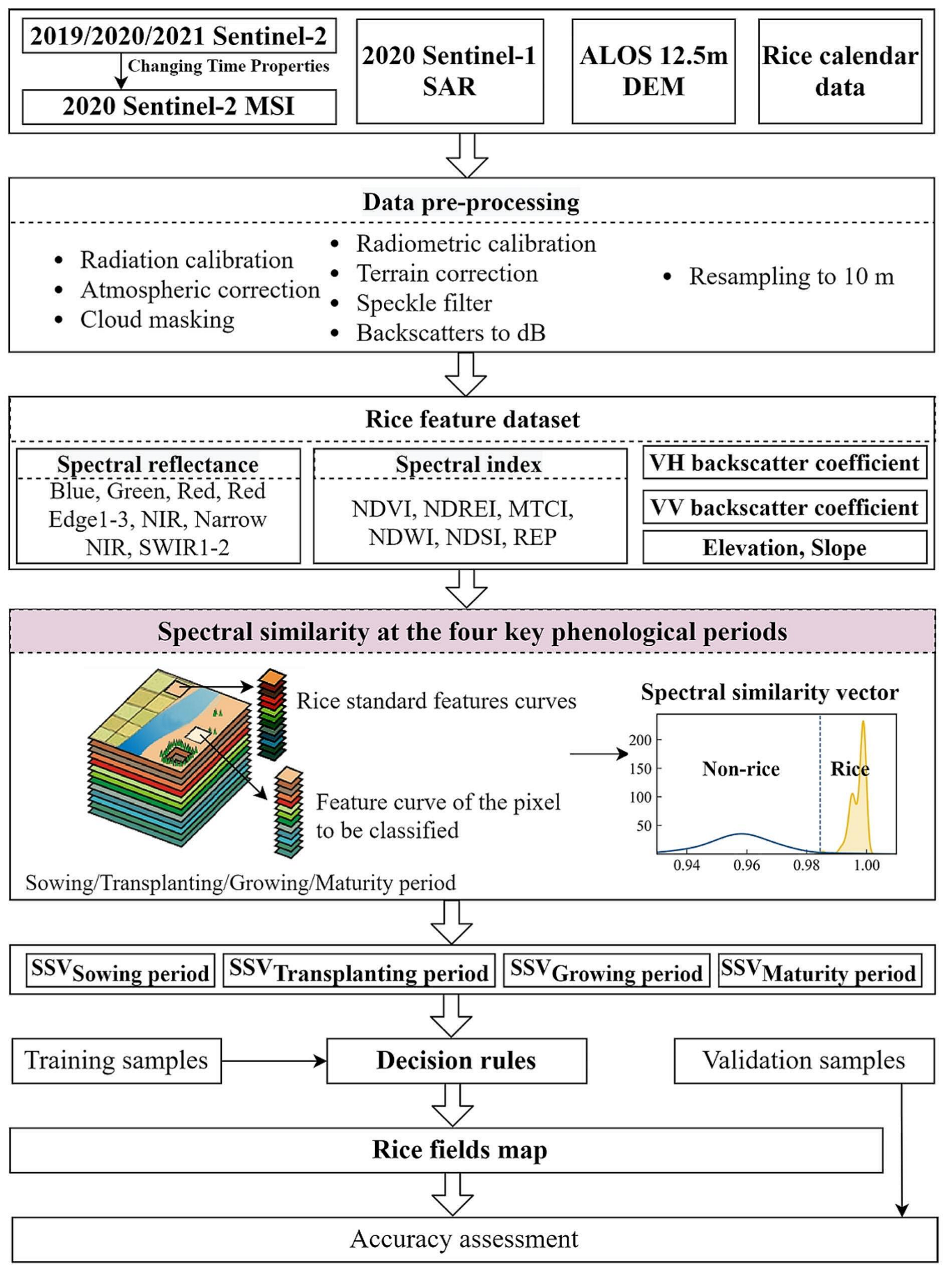
The solution for high-precision identification of rice fields in hilly areas based on the Sentinel-1 SAR, Sentinel-2 MSI, DEM, and rice calendar data

For high-precision recognition of paddy fields in hilly regions, we proposed a solution based on the Sentinel-2 MSI, Sentinel-1 SAR, DEM, and rice calendar data (Fig. 2). The core content included the construction of rice feature dataset, the generation of rice standard feature curves, and the proposal of the SSV algorithm for rice identification. Specifically, based on data preprocessing, we constructed a rice feature dataset with a total of 24 features in three categories, (optical features, SAR features, and topographic features), including 10 time-series rice spectral reflectance, 10 time-series rice spectral indices, 2 time-series VH and VV backscatter coefficients, and 2 topographic features. Then, we generated the standard feature curves of rice at the four key phenological periods (sowing, transplanting, growing, maturity). Furthermore, we proposed the SSV algorithm to measure the similarity between unknown pixels and rice standard feature curves for rice identification. High-precision identification and extraction of rice fields were achieved based on their similarity values. As for the accuracy evaluation, the rice validation samples and interpretation results from Unmanned Aerial Vehicle (UAV) data were utilized.

### Rice feature dataset

#### Optical features

The optical features include the spectral reflectance of Sentinel-2 in 10 bands (Blue, Green, Red, Red Edge1-3, NIR, Narrow Nir, SWIR1-2) and 6 spectral indices calculated based on the reflectance bands. Spectral indices include 1 vegetation indice (NDVI), 1 water indices (NDWI), 2 red-edge indices (Normalized Difference Red Edge Index (NDREI), Red-Edge Position Index (REP)), 1 Medium Resolution Imaging Spectrometer terrestrial chlorophyll index (MTCI) and 1 Normalized Difference Soil Index (NDSI) [22].

Rice has high reflectance in red-edge and infrared bands, which is higher than that in green, blue, and red bands. The NIR band is not sensitive to changes in water content in rice fields or rice canopies, while the SWIR is sensitive. NDVI index can be better used to express changes in greenness of rice. The MTCI can reflect the chlorophyll content of the rice canopy by combining visible and near-infrared bands. NDSI is also sensitive to the moisture content of the rice field and is positively correlated with the water content. There is a monotonic relationship between NDREI, REP and chlorophyll concentration in the rice canopy.

**Fig. 3:**
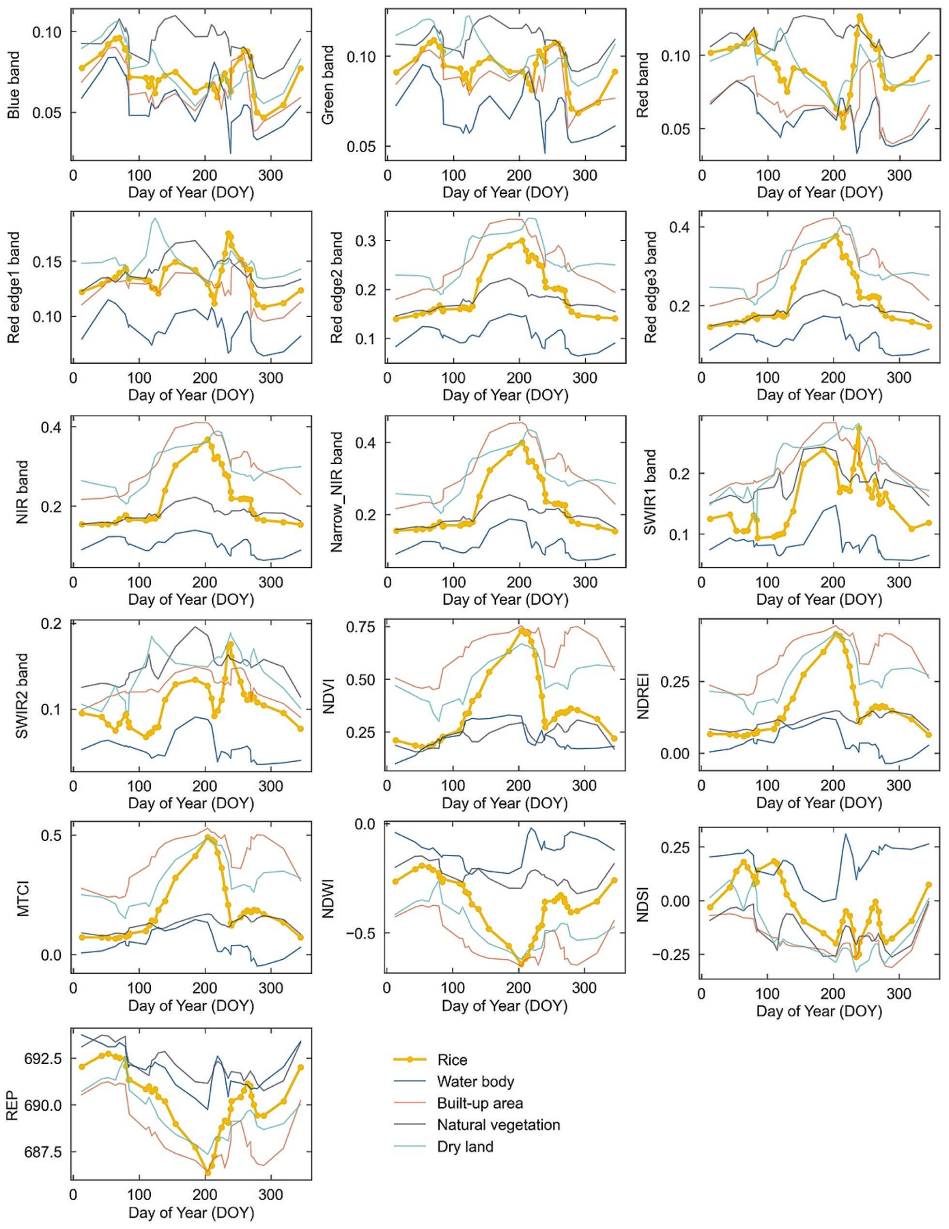
Time series spectral reflectance and spectral indices of major land cover types

During the sowing period, before transplanting (DOY60-110), the water content of the rice field increased due to farmers flooding the rice field. At this time, the reflectance in each band was low (Fig. 3). NDVI, NDREI and MTCI were small. NDWI was large and showed an increasing trend. During the transplanting period (DOY110-130), due to the small rice nursery plants and their sparsely distributed, the paddy fields were still mainly covered by water bodies, the reflectance and vegetation indices were still low, NDWI values remained high. During the growing period (DOY130-230), the water content of rice fields gradually decreased, the chlorophyll content of rice plants gradually increased. This led to an increasing trend of vegetation indices, NDREI, and MTCI, which were positively correlated with vegetation cover and chlorophyll content. On the contrary, water indices REP, and NDSI presented a decreasing trend. As the rice matured, the leaves began to wilt and become yellow, the chlorophyll content correspondingly decreased, so the green-band reflectance, NDVI, NDREI, and MTCI began to decrease.

#### SAR features

**Fig. 4:**
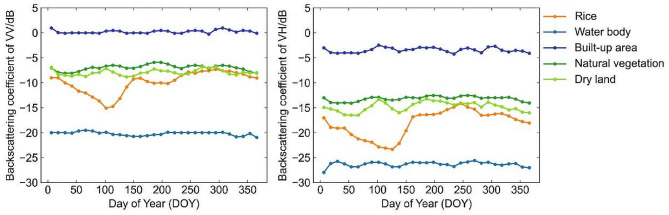
Time series VV and VH backscatter coefficients for rice and other major land cover types

During the rice phenological period (DOY60-270), the trend of VV backscatter coefficient of rice decreased, increased, then slightly decreased, and increased again. The trend of VH backscatter coefficient of rice changed from decreasing to increasing, and then decreasing (Fig. 4). During the sowing period (DOY60-110), farmers flooded the rice fields. The scattering mechanism of rice field was mainly controlled by specular reflection from smooth surfaces. The backward scattering coefficient was low and decreased. At the transplanting stage (DOY110-130), rice nursery plants were short and sparse. The scattering mechanism of the paddy field was mostly determined by surface scattering resulted from water bodies and moist soil, with little body and secondary scattering. The backscattering coefficient of rice field was still low at this time, the VH backscattering coefficient was accordingly < -20 dB. At the growth period (DOY130-230), the number of rice tillers increased, the stem length became longer, and the leaves developed fully and contributed to a denser canopy of rice. As the rice entered the spike stage, the spikes straightened and had no sign of bending. The plants grew to their maximum height and developed a closed canopy. The backscattering mechanism in rice fields at this time was mainly body scattering caused by rice structure, with little surface and secondary scattering. The backscattering coefficient of rice during the growing period tended to increase, with the VH backscattering coefficient increasing from less than − 20 dB to -15 dB. At maturity (DOY230-270), rice gradually changed from lime green to yellow, and the biomass decreased. The backscattering coefficient accordingly gradually decreased. After rice harvest, the rice field was covered by straw or had a water-bearing layer, the backscattering coefficient correspondingly decreased again.

#### Terrain features

A single rice field is relatively flat to facilitate water storage for rice growth. Under complex topographic conditions, rice fields are more fragmented. Therefore, we considered slope information in topographic factors to improve rice identification accuracy.

### Rice standard features curves

**Fig. 5:**
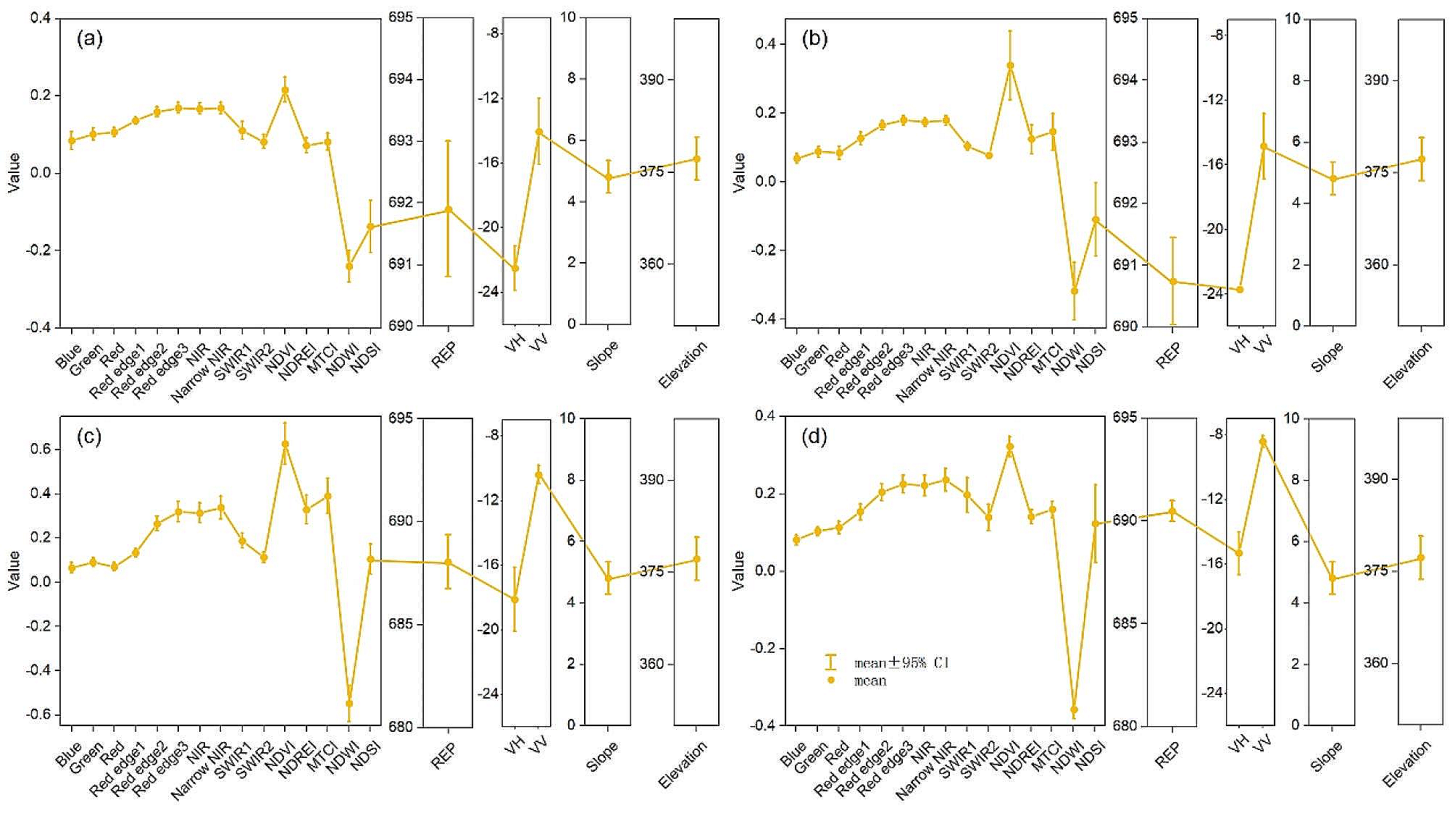
Standard features curves of rice at **(a)** sowing, **(b)** transplanting, **(c)** growing and **(d)** maturity stages

By utilizing the mean composite technique, we calculated the average optical and SAR feature values for each of the four primary phenological periods, and combined these features to produce a multi-band feature image. Then the standard feature curves of rice at each of the four primary phenological periods were generated based on the rice samples (Fig. 5). The standard feature curve of rice is the mean value of the feature curves of these rice sample points.

### Spectral similarity vector

**Fig. 6:**
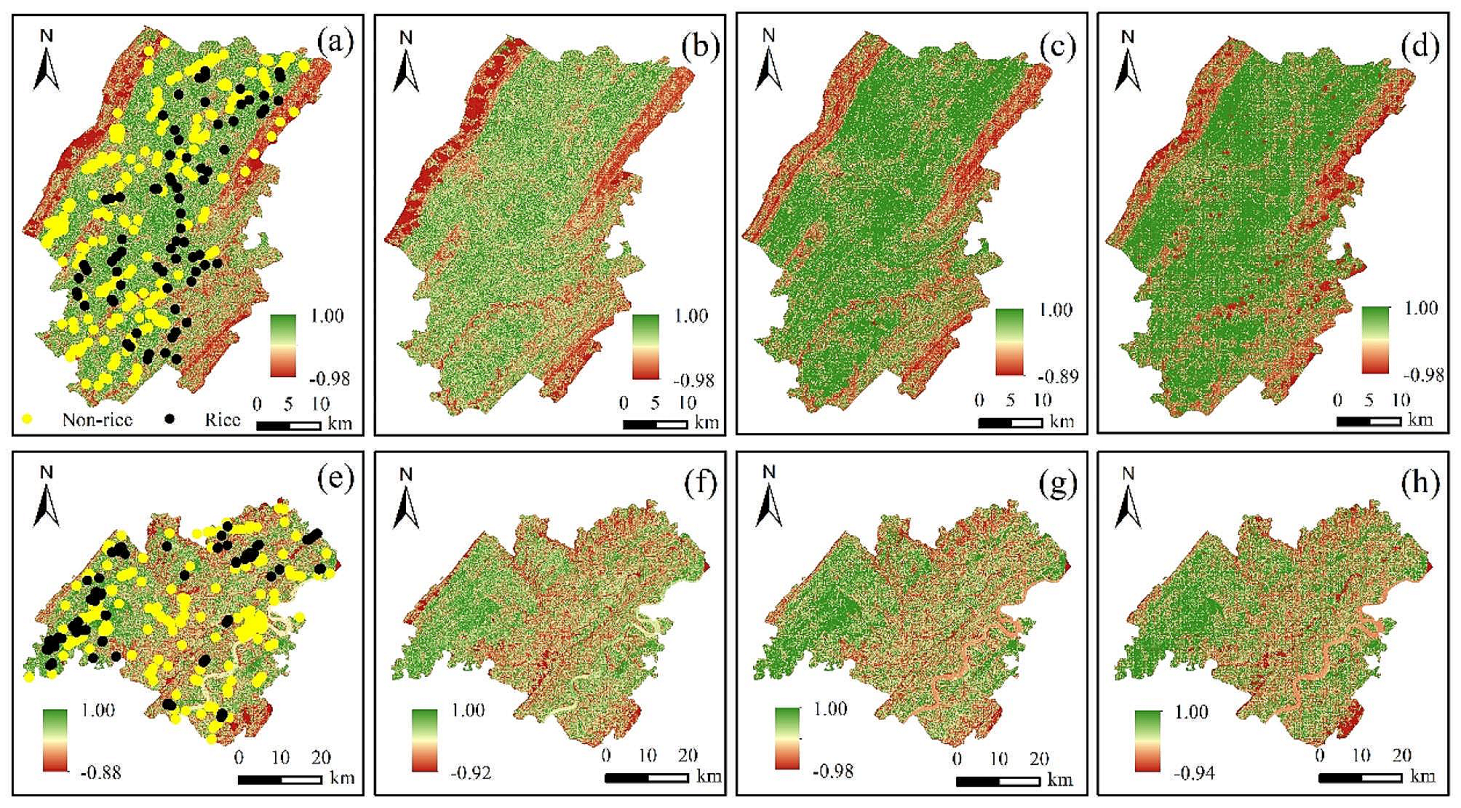
Spectral similarity images of four key phenological periods, the first row is for Dianjiang County, the second row is for Zhongxian County, and the first to fourth columns correspond to the sowing, transplanting, growing and maturing periods, respectively

Euclidean distance can measure the closeness between the feature curve of the image element to be classified and the standard feature curve of rice [38]. Cosine similarity can evaluate the similarity of two vectors by the cosine of their angle [64]. Matched with the feature vectors of standard rice pixels, the cosine similarity of other rice pixels is greater than that of non-rice pixels; The Euclidean distance of other rice pixels is smaller than that of non-rice pixels. Therefore, the difference between cosine similarity and Euclidean distance can expand the difference between rice and non-rice, facilitate the determination of thresholds, and improve classification accuracy. We therefore proposed the Spectral similarity vector (SSV) algorithm to measure the similarity between unknown pixels and rice standard feature curves for rice identification, which were based on multi-band feature datasets and rice standard feature curves at four key phenological periods (Fig. 6). The specific formula expression is as follows.

The Euclidian distance is defined as:

1 $$\begin{array}{*{20}{c}}{d(x,y) = \sqrt {\sum\limits_{i = 1}^n {{{({x_i} - {y_i})}^2}} } } \\ {d{{(x,y)}_{norm}} = (d - {{\text{d}}_{min}})/({d_{max}} - {d_{min}})} \end{array}$$

where *x*, *y* denotes the feature vectors (feature curve values) of the pixel to be classified and the standard rice, respectively. *n* is the number of features, $$ d(x, y)$$ is the original Euclidean distance, and $$ {d(x, y)}_{norm}$$ is the normalized Euclidean distance, the $$ {d}_{max}$$ and $$ {d}_{min}$$ refers to the maximum and minimum pixel values in Euclidean distance image.

The cosine similarity (CS) is defined as:

2 $$CS(x,y) = \frac{{\sum\nolimits_{i = 1}^n {\left( {{x_i}{y_i}} \right)} }}{{\sqrt {\sum\nolimits_i^n {{{\left( {{x_i}} \right)}^2}} } \sqrt {\sum\nolimits_i^n {{{\left( {{y_i}} \right)}^2}} } }}$$

Spectral similarity vector (SSV) is defined as:

3 $$ SSV=CS(x,y)-{d(x, y)}_{norm}$$

### Decision rules for rice identification

**Fig. 7:**
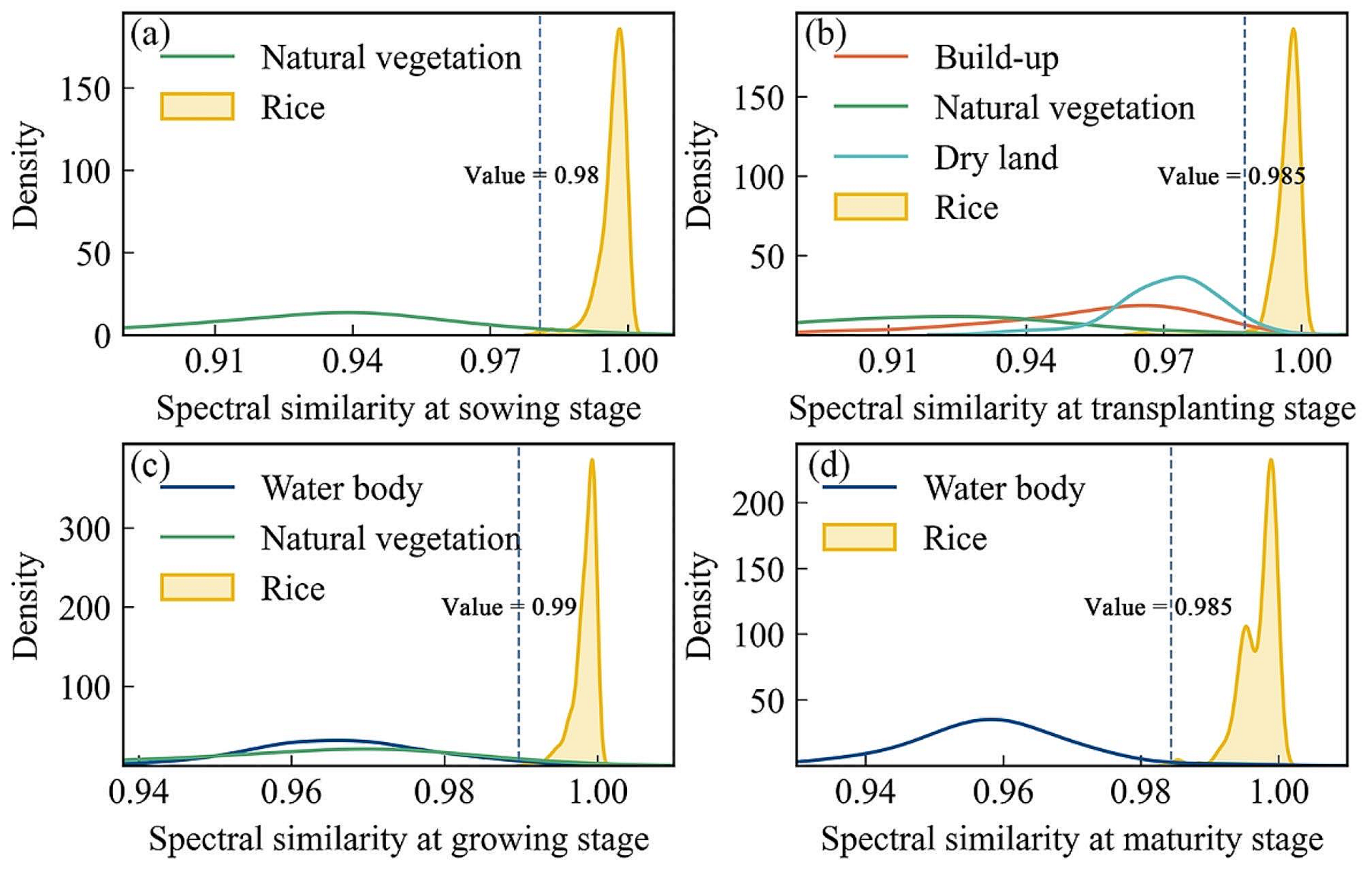
Histograms of spectral similarities between rice and other land cover types and thresholds for identifying rice

Based on the spectral similarity plots for the four phenological periods as shown in Fig. 6 and the sample points for rice and non-rice, histograms were produced as shown in Fig. 7. The threshold for distinguishing between rice and non-rice areas is determined based on Spectral similarity vector (SSV) and sample points. Specifically, firstly, the spectral similarity values of the feature curves of the rice and non-rice samples with the rice standard feature curves are calculated separately; secondly, the 25% quartile of the spectral similarity values corresponding to the rice samples and the 75% quartile of the spectral similarity values corresponding to the non-rice samples are taken; and lastly, the mean value of the two is taken to be the optimal threshold for distinguishing between rice and non-rice. It can be calculated that at the sowing stage, a threshold of 0.98 is appropriate for distinguishing between rice and natural vegetation based on spectral similarity. Similarly, at the transplanting stage, a threshold of 0.985 is suitable for distinguishing between rice and build-up, natural vegetation, and dry land. Moreover, during the growing stage, a threshold of 0.99 is appropriate for distinguishing between rice and water bodies, as well as natural vegetation. Finally, at the maturity stage, a threshold of 0.99 is suitable for distinguishing between rice and water bodies.

Based on the above analysis, the constructed rice identification algorithm integrating SSV and decision rules is as follows:

*if SSV*_*Sowing stage*_≥0.98 *and SSV*_*Transplanting stage*_≥0.985 *and SSV*_*Growing stage*_ ≥ 0.99 and *SSV*_*Maturity stage*_≥0.985,

*Value*_*Land*_=1,

*else Value*_*Land*_ =0,

*end*.

where *Value*_*Land*_ equals 1 for rice and *Value*_*Land*_ equals 0 for non-rice.

## Results

### Rice fields recognition

**Fig. 8:**
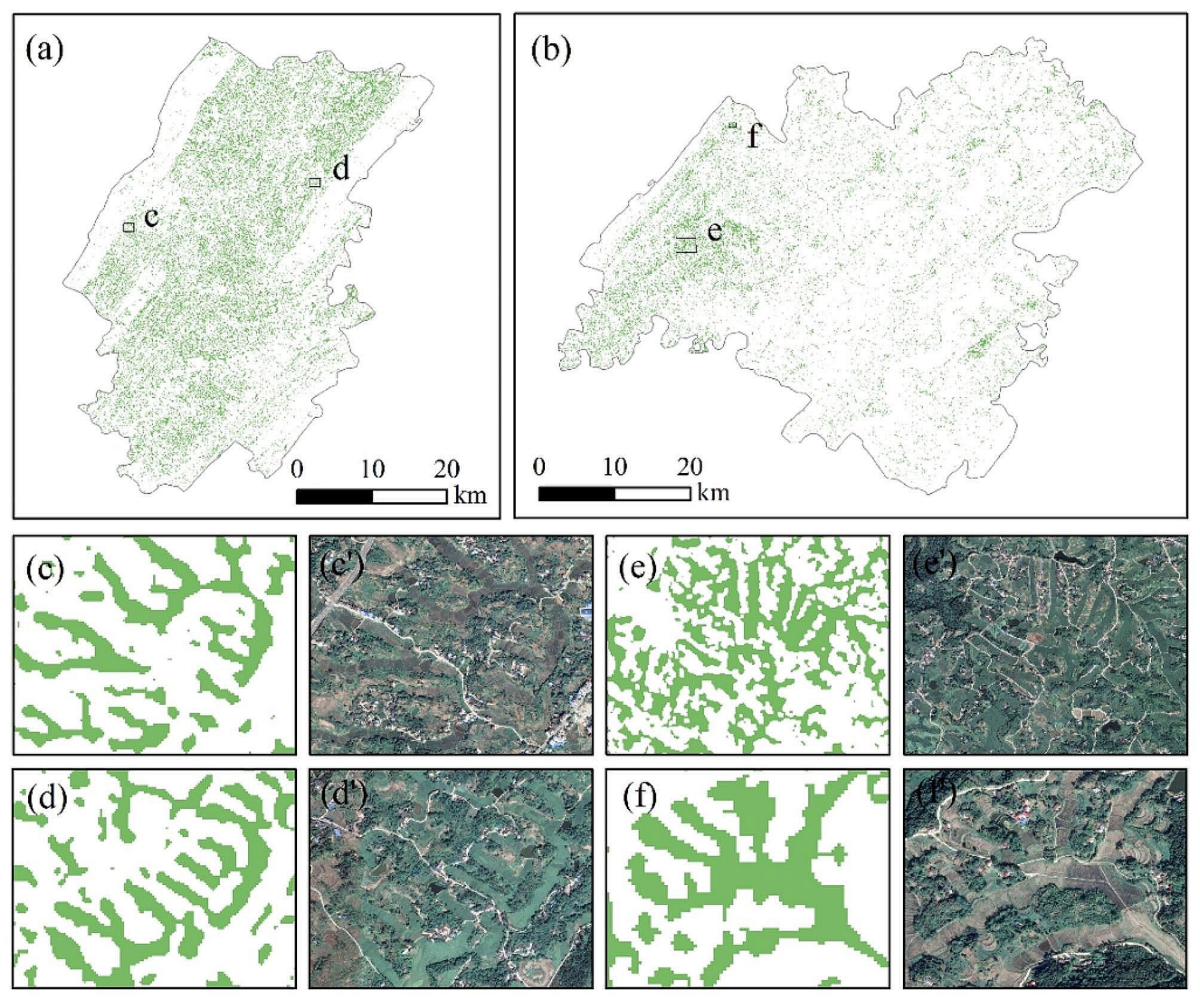
The paddy rice map of Dianjiang county **(a)**, Zhongxian county **(b)** and the magnified view of four small areas

The rice distribution maps obtained from the proposed solution based on the multi-source remote sensing for Dianjiang and Zhongxian counties were shown in Fig. 8. The identified rice areas of Dianjiang and Zhongxian counties in 2020 were 254.04 km^2^ and 205.03 km^2^, respectively. The spatial distribution of rice in Dianjiang County was more uniform with hilly and flat dam areas dominating. Rice cultivation in Zhongxian County was mainly concentrated in the low mountainous and western hilly regions, while rice was sparsely distributed in the high mountainous areas in the central and east. The rice identification results of four small randomly selected areas (c, d, e, f) were in good agreement with the high-resolution Google Earth images.

### Rice fields recognition accuracy

The accuracy of rice field recognition was analyzed using the validation samples, and the results were presented in Table 3. The user accuracy, producer accuracy, overall accuracy, and kappa of rice identification in Dianjiang County were 0.95, 0.94, 0.96 and 0.92, respectively. Those in Zhongxian County were 0.92, 0.85, 0.88 and 0.78, respectively. All accuracy indicators in both experimental areas were above 0.85, which suggested that the proposed solution demonstrated its effectiveness in accurately identifying rice and effectively distinguishing between rice and non-rice land cover types.

**Table 3:** Accuracy assessment of rice map in Dianjiang and Zhongxian County based on the validation samples

Case study areas	Land cover types	Rice	Non-rice	User accuracy	Producer accuracy	Overall accuracy	Kappa coefficient
Dianjiang County	Rice	61	3	0.95	0.94	0.96	0.92
	Non-rice	4	118	0.97	0.96		
Zhongxian County	Rice	34	3	0.92	0.85	0.88	0.78
	Non-rice	6	37	0.86	0.93		

## Discussion

### Assessment of the solution’s ability for different sized rice fields

**Fig. 9:**
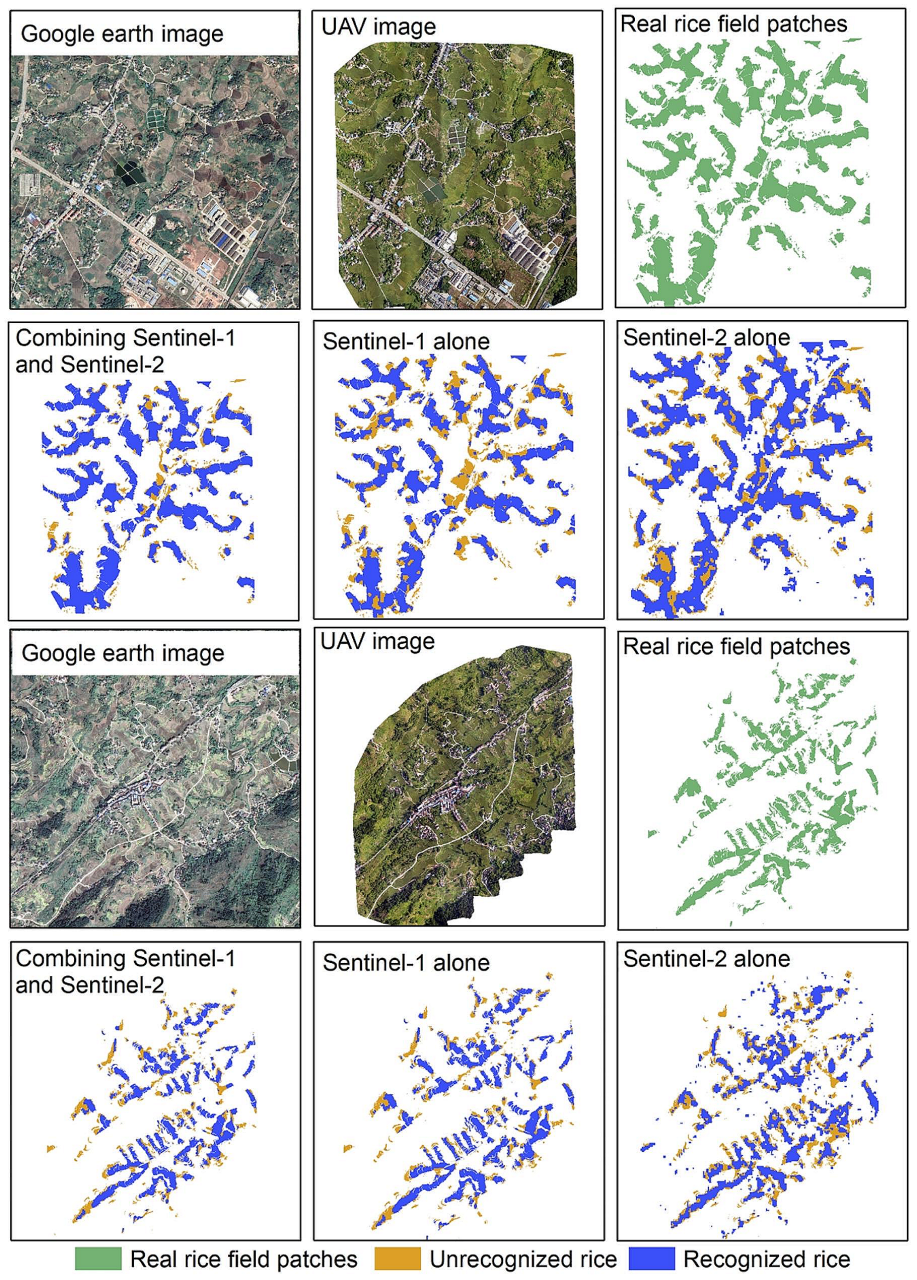
Rice identification results when using Sentinel-1 and Sentinel-2 combined versus Sentinel-1 alone or Sentinel-2 alone

**Fig. 10:**
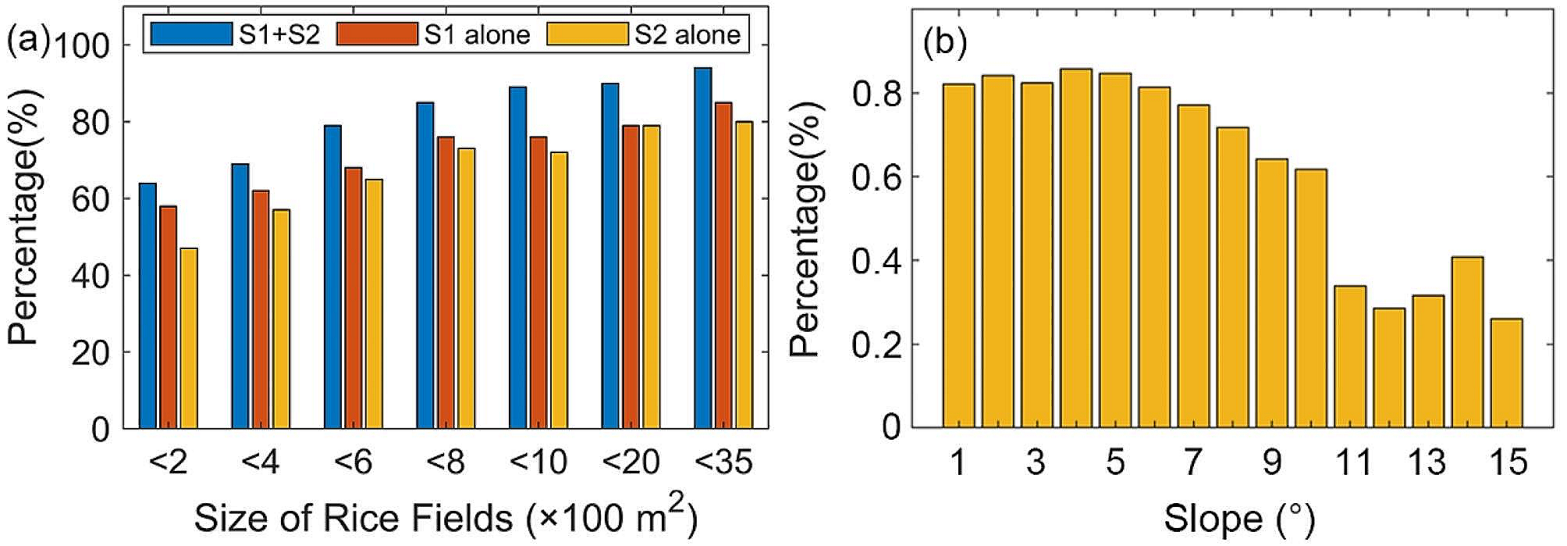
Proportions of recognized pixels with the changes of rice field sizes **(a)**. Proportions of recognized pixels with the changes of rice field slope **(b)**

A more accurate spatial distribution of rice fields was obtained based on high spatial resolution UAV images, which was employed to quantitatively evaluate the omission error of SSV-based rice field identification results (Fig. 9). The interpretation results from UAV were marked as the recognized and unrecognized rice pixels. The proportion of recognized and unrecognized rice pixels with the change in rice field sizes was presented in Fig. 10a. The results showed that when the rice field area is more than 400 m^2^ (equivalent to 4 pixels), more than 79% of rice fields are successfully identified. And this percentage can achieve 89% when the rice field area is larger than 800 m^2^.

To validate the capability of the proposed solution based on multi-source remote sensing in different rice paddy patches, rice identification based on single Sentinel-1 SAR and single Sentinel-2 MSI were simultaneously implemented, respectively (Figs. 9 and 10a). The findings indicated that using SAR and MSI in conjunction led to a higher recognition proportion of rice, with an improvement of at least 8% over using only SAR or MSI in all area intervals, especially the 400 m^2^ to 2000 m^2^ area interval. The proportion of rice that was identified solely by either SAR or MSI in this interval was less than 0.8, whereas the proportion of rice identified by the combination of SAR and MSI was more than 0.8. Therefore, it can be speculated that even with the combination of SAR and MSI, the identification proportion of small-sized (less than 400 m^2^) rice fields remains relatively low. This indicated that the combined SAR and optical can significantly improve the recognition ratio for rice fields with areas between 400 m^2^ and 2000 m^2^.

### Assessment of the solution’s ability for rice fields at different slopes

To evaluate the algorithm’s ability to identify rice at different slopes, we measured the ratio of successfully recognized rice area to the total area across various slope intervals (Fig. 10b). The findings revealed that the algorithm could identify over 80% of rice fields with slopes less than 6°. For rice fields with slopes from 6° to 10°, the recognition rate ranged from 62 to 77%. However, the algorithm struggled to recognize rice fields with slopes exceeding 10°. This was primarily because such fields were predominantly terraced and had small areas, with the 25th and 75th percentiles of area measuring 35.22 m^2^ and 285.07 m^2^, respectively. Regarding the imaging mechanism, secondary scattering between the terraced fields and fields levees in SAR images led to abnormal large SAR backscattering coefficients. In optical images, more reflectivity information from the narrow fields levees was captured in the same image element. As a result, the information about rice on narrow and small terraces was not adequately represented in both the 10-m spatial resolution SAR image and the optical image. To accurately identify rice planted on hillsides with slopes exceeding 10°, higher spatial resolution remote sensing images were needed.

### Comparison of rice identification capabilities among different data sources

Table 4 presented the results of different remote sensing sources on the accuracy of rice identification. The findings indicated that the highest accuracy in identifying rice was achieved when SAR and MSI were combined. When only Sentinel-1 was used, the overall accuracy of the two study areas decreased by 0.04 (from 0.96 to 0.92) and 0.01 (from 0.88 to 0.87) respectively. Similarly, when only Sentinel-2 was used, the overall accuracy decreased by 0.05 (from 0.96 to 0.91) and 0.02 (from 0.88 to 0.86) respectively. Additionally, the user accuracy decreased by 0.16 (from 0.95 to 0.79) and 0.09 (from 0.92 to 0.83) respectively.

**Table 4:** The performance of rice mapping with multi-remote sensing data versus rice mapping with single remote sensing data

Remote sensing data	Accuracy	Dianjiang County	Zhongxian County
Combining Sentinel-1 and Sentinel-2	UA	0.95	0.92
	PA	0.94	0.85
	OA	0.96	0.88
	Kappa	0.92	0.78
Seninel-1 alone	UA	0.82	0.85
	PA	0.96	0.89
	OA	0.92	0.87
	Kappa	0.81	0.75
Seninel-2 alone	UA	0.79	0.83
	PA	0.95	0.92
	OA	0.91	0.86
	Kappa	0.79	0.73

When optical remote sensing data are available, the combined use of optical and SAR can improve rice identification accuracy. This is because optical remote sensing has higher spatial resolution and can provide more detailed information on the ground surface. Optical remote sensing observation of rice relies on the interaction between rice and electromagnetic waves, especially the absorption and reflection of electromagnetic waves by the canopy of rice plants can directly reflect the spectral characteristics of rice, which has obvious advantages [47]. Before rice transplanting, the corresponding image elements in rice fields reflect the spectral characteristics of wet bare soil or shallow standing water. As rice grows and develops, the canopy cover of rice plants and chlorophyll content increases, more leaves are involved in photosynthesis, and the biomass is high, reflecting more of the spectral characteristics of vegetation [16]. For Sentinel-1 SAR data, it can provide dual-polarized backscattering information sensitive to water content and roughness of rice fields. Also, it can be imaged continuously and stably without the influence of solar radiation and clouds, making the time-series backscatter information a good and complete characterization of rice phenology. Moreover, the mean-composite Sentinel-1 images include images with ‘ASCENDING’ or ‘DESCENDING’ orbitProperties. Therefore, the mean composite for both ascending and descending Sentinel-1 images naturally suppresses backscatter intensity anomalies caused by foreshortening and shadowing, thereby improving image quality and rice field identification accuracy.

## Conclusions

Considering the different imaging mechanisms and wavelength ranges of optical and SAR images, we combined the respective advantages of optical and SAR to focus on the identification of rice fields in hilly areas, aiming to achieve high-precision recognition of small- and medium-sized rice fields with fragmented and dispersed distribution. We therefore proposed a solution on the basis of the Sentinel-2 MSI, Sentinel-1 SAR, DEM, and rice calendar data. This solution mainly included the construction of rice feature dataset at four crucial growth stages, the generation of rice standard spectral curve, and the proposal of spectral similarity algorithm for rice identification. The proposed solution manifested its effectiveness in identifying rice fields in hilly areas with the overall accuracy exceeding 0.85. Meanwhile, the study assessed the recognition capability of the proposed solution for rice paddy fields of different patch sizes and varying terrains. It also revealed that the synergy of Sentinel-1 SAR and Sentinel-2 MSI data significantly enhanced the recognition ability of rice paddy fields ranging from 400 m^2^ to 2000 m^2^. In future studies, efforts to improve the algorithm should concentrate on the automatic construction of rice feature dataset and adaptive determination of similarity threshold.

## Data Availability

Sentinel-1/2 and elevation data used in this study are freely available. Rice calendar data were provided by Chongqing Meteorological Bureau. Anyone who wants to use the data can contact the corresponding author Lihua Wang (e-mail: wanglihua1@nbu.edu.cn).
